# Experimental study of the bilateral asymmetric single-rivet occluder device for transcatheter patent foramen ovale closure with reserved interatrial septal puncture area

**DOI:** 10.3389/fcvm.2023.1301412

**Published:** 2024-01-05

**Authors:** Yiting Wei, Yanlin Su, Suyan Cao, Ziqian He, Renrong Wang, Xian Qin, Yuanxi Feng, Chengjian Yang, Haibin Jiang

**Affiliations:** Department of Cardiology, Wuxi No.2 People's Hospital (Jiangnan University Medical Center), Wuxi, Jiangsu, China

**Keywords:** interventional catheterization, congenital heart disease (CHD), patent foramen ovale (PFO), occluder, atrial septal puncture

## Abstract

**Purpose:**

To evaluate a noval bilateral asymmetric single-rivet occluder with reserved interatrial septal puncture area for treating patent foramen ovale (PFO).

**Materials and methods:**

The study established a pig model of patent foramen ovale (PFO) by puncturing the oval fossa and then performing high-pressure balloon dilation. A specially designed bilateral asymmetric occluder for the reserved interatrial septal puncture area was then. used to close the PFO through catheter-based intervention. The pigs were kept for 3 months before undergoing a second catheter-based intervention, involving interatrial septal puncture using a newly developed occluder in the reserved interatrial septal puncture area. During 6 months, the experimental pigs underwent assessment using digital subtraction angiography (DSA), echocardiography, and histological evaluation.

**Results:**

A patent foramen ovale (PFO) model was successfully established in 6 pigs using the puncture atrial septum high-pressure balloon dilation method. The diameter of the unclosed PFO was measured (3.56 ± 0.25 mm). Using the newly developed occluder device, all 6 pigs with unclosed PFO underwent successful catheter-based closure surgeries, with intraoperative and postoperative transesophageal echocardiography showing excellent device positioning and complete closure without residual shunting. After 3 months of implantation, the catheter-based interatrial septal puncture was performed through the reserved interatrial septal puncture area, and all procedures were successful. Immediately following euthanasia, a histological examination revealed intact and undamaged occluder devices with visible puncture holes in the reserved interatrial septal puncture area. No fracture of the nitinol wire was observed, and the surface of the occluder device showed coverage of endothelial and connective tissues. Utilizing a bilateral asymmetric single-rivet occluder device implanted through the reserved interatrial septal puncture area has proven effective in closing PFO. After implantation, the occluder device allows subsequent interatrial septal puncture procedures through the reserved area.

**Conclusion:**

The novel occluder device demonstrated excellent closure performance, biocompatibility, and puncturability in the experiment. This indicates the feasibility of conducting further catheter-based interventions on the interatrial septum.

## Introduction

Patent foramen ovale (PFO) can lead to cryptogenic stroke, ischemic stroke, migraine, and other diseases, with a prevalence rate of 20%–30% even in the general population (1, 2). Currently, pharmacotherapy utilizing antiplatelet or anticoagulant medications is employed for treating PFO. Additionally, interventional treatment approaches encompass a range of surgical devices. Among them, the most common is the nickel-titanium PFO occluder device series represented by the Amplatzer® occluder (ADO) (St. Jude Medical, Inc.; St. Paul, Minnesota).

However, the clinical septal occluder devices currently being used encounter difficulties in establishing a puncture path through the atrial septum after closing the PFO. This is primarily due to the presence of metal discs on both sides of the occluder device. With the progress of population aging, there has been a rise in the incidence of atrial fibrillation and valve diseases. Minimally invasive procedures such as pulmonary vein isolation for atrial fibrillation, left atrial appendage closure, and transcatheter mitral valve replacement require interatrial septal puncture. However, the presence of occluder devices poses obstacles to transseptal interventions. To facilitate interatrial septal puncture and subsequent minimally invasive interventions after PFO closure, we have designed a symmetric residual puncture area PFO occluder device, demonstrating good performance in experimental studies (citation). In the present study, we further designed an asymmetric nickel-titanium occluder device (Shanghai Push Medical Device CO., LTD.) with a reserved asymmetric puncture area in the septum. After verifying its effectiveness in closing the PFO, we validated its ability to facilitate interatrial septal puncture through the reserved puncture area on the occluder device.

## Materials and methods

### Device and laboratory animals

**Figure 1:**
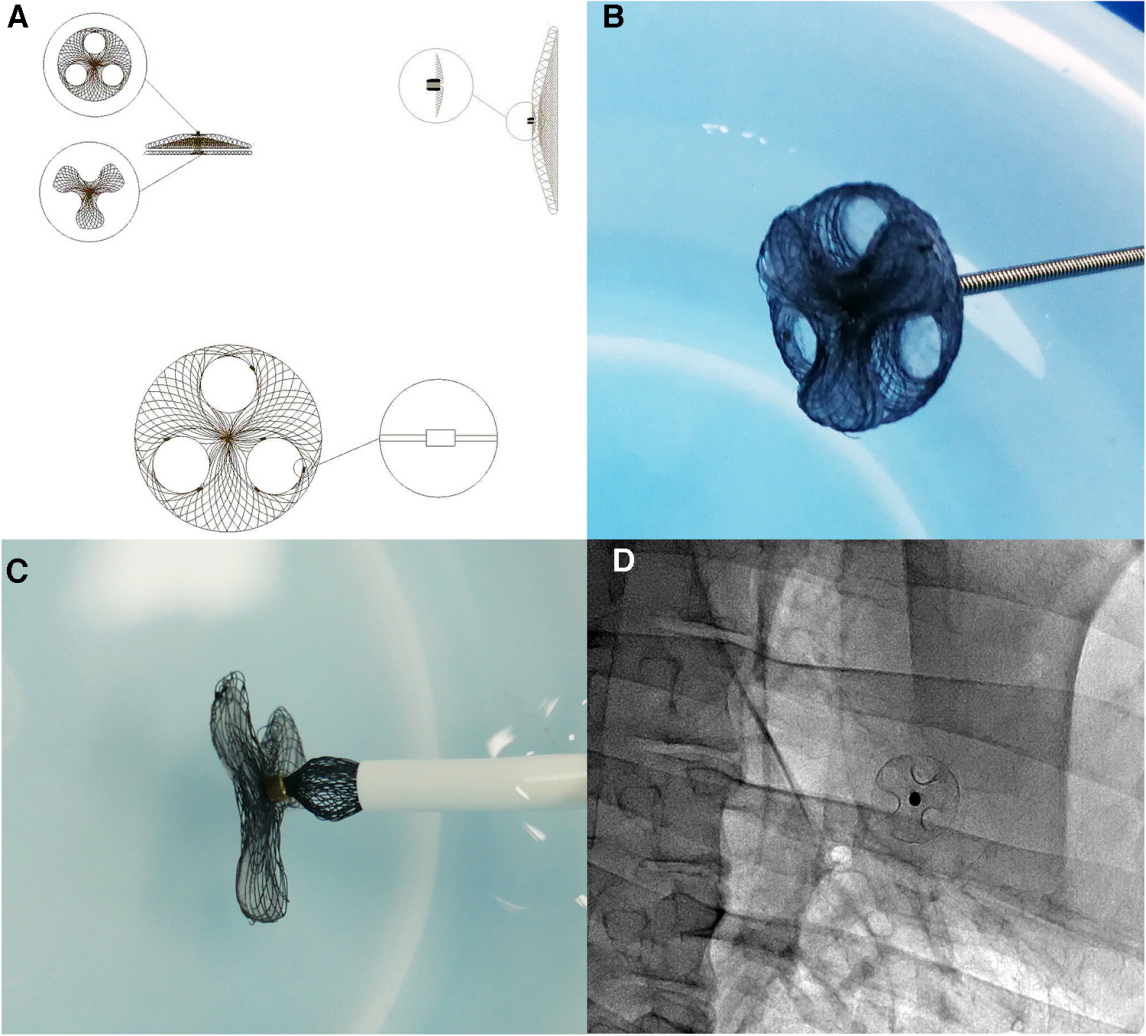
Structure and morphology of the asymmetric single-wire PFO occluder device with a reserved interatrial septal puncture area: (**A**) design diagram of the asymmetric single-wire PFO occluder device with a reserved interatrial septal puncture area. The right atrial side of the occluder device is a circular metal disc with evenly distributed circular holes for the reserved interatrial septal puncture area. Tantalum particles with stronger imaging capability are embedded along the edges of the reserved interatrial septal puncture holes, serving as markers for interatrial septal puncture under DSA. (**B**) Photograph of the asymmetric single-wire PFO occluder device with a reserved interatrial septal puncture area. (**C**) Insertion of the right atrial side of the asymmetric single-rivet PFO occluder device with a reserved interatrial septal puncture area into the sheath. (**D**) DSA image after implantation of the asymmetric single-rivet PFO occluder device with a reserved interatrial septal puncture area in the body.

The main body of the asymmetric single-wire PFO occluder device with a reserved interatrial septal puncture area is made of superelastic nickel-titanium wire. The right atrial surface forms a disc-shaped metal disc, with a stainless steel rivet at the center to fix the main body of the occluder device and connect the delivery cable. On the inner side of the right disc, there are three layers of biodegradable polyester fiber membrane to block shunting. The reserved interatrial septal puncture area is evenly distributed around the disc, where the nickel-titanium wire weaving structure is absent. Instead, only a biodegradable blocking membrane is set. The left disc on the left atrial side is densely woven with nickel-titanium wires, forming a three-leaflet petal shape. There is no fixed steel ring in the center and no obstructive polyester fiber membrane inside. The occluder device can be delivered through a delivery sheath ranging from 8 F to 12 F (Figure 1).

Method of establishing an artificial PFO model by high-pressure balloon dilation of the interatrial septum puncture site. A total of 6 experimental pigs were used to establish an animal model of PFO: (1) Preoperative preparation was conducted on the experimental animals three days before surgery, with fasting but free access to water for 72 h before the procedure. (2) Water was withheld on the morning of the surgery. (3) Anesthesia was induced by intramuscular injection with Tiletamine hydrochloride and Zolazepam hydrochloride. After successful induction of anesthesia, tracheal intubation was then performed to establish a respiratory pathway, and sevoflurane was inhaled for anesthesia maintenance. Venous access was established by puncturing the jugular vein, and arterial access was obtained by puncturing the femoral artery for dynamic blood pressure monitoring using a vascular sheath. The experimental animals were placed in a supine position and fixed to a specially designed bracket on the surgical table, with electrocardiographic monitoring throughout the procedure.

### Establishing patent foramen ovale animal models

The procedure involved the following steps: placing a sterile drape, puncturing the right femoral vein, inserting a 7The occluder device can be delivered through a delivery sheath ranging from 8F to 12FF vascular sheath, and administering 2,000 U of heparin through the vein. Under electrocardiographic monitoring, right heart catheterization was performed to assess pressures in the right atrium, right ventricle, and pulmonary artery. Transthoracic echocardiography was used to visualize both atria and determine the position of the interatrial septum and superior vena cava. Under fluoroscopic guidance, an 8The occluder device can be delivered through a delivery sheath ranging from 8F to 12FF interatrial septal puncture sheath was advanced from the femoral vein using a 0.032-inch guidewire, up to the superior vena cava. The Swartz catheter was utilized according to the three-point method for interatrial septal puncture of the oval fossa: ① The tip of the catheter core is slowly slid downward to the 4 o'clock position and fixed at the location of the oval fossa. ② Rotate 90° to the left lateral position, align the direction of the catheter core tip parallel to the spine, positioned between the midpoint of the posterior border of the cardiac silhouette and the line connecting to the aorta, approximately one-third from the middle to the posterior. The sheath slides upward, reaching into the oval fossa. Advance the catheter core, feeling a breakthrough and monitoring the pressure, then inject a contrast agent into the left atrium after confirming left atrial pressure, observing the dispersion of the contrast agent in the left atrium. Continue advancing the sheath while penetrating through the oval fossa. ③ Rotate 30° to the right anterior oblique position, fix the catheter core, gently insert the Swartz catheter, and secure the inner catheter before approaching the ascending aorta. Continue pushing the outer sheath, causing the distal end of the outer sheath to bend beneath the aortic valve. Withdraw the catheter core, insert a guidewire into the left atrium through the Swartz catheter, and introduce the DKonquer balloon (DK Medtech, Inc; Suzhou Jiangsu) with a diameter of 5 mm into the left atrium, repeatedly dilating the oval fossa to create a certain diameter PFO model.

### Closure of artificial patent foramen ovale with reserved atrial septal puncture area

After measuring the diameter and morphology of the artificial PFO using transesophageal echocardiography, exchange for a stiffer guidewire, and successfully close the PFO model according to the standard procedure for PFO closure. Postoperatively, perform electrocardiogram and transesophageal ultrasound to observe the closure status, presence of shunting, and possible arrhythmias. During and after the procedure, administer an intramuscular injection of 100,000 U/kg penicillin for infection prevention, and continue penicillin treatment for 3 days.

### Transarterial septal puncture through reserved atrial septalpuncture area device

**Figure 2:**
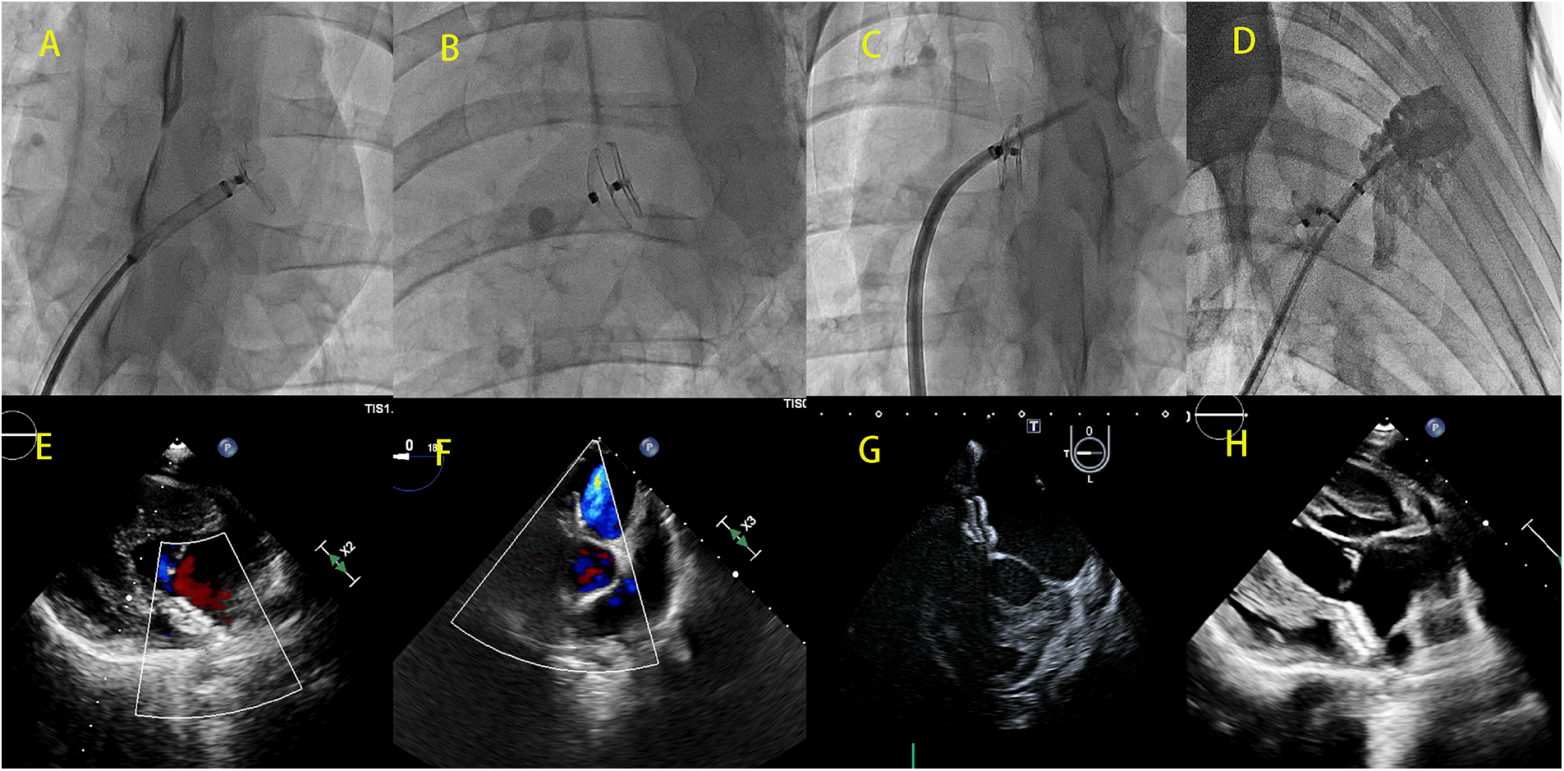
DSA images (**A–D**) and cardiac ultrasound images (**E–H**) showing the implantation of a bilateral asymmetric single-rivet PFO occluder device in the reserved interatrial septum puncture area. (**A**) Left ventricular disc of the deployed occluder device; (**B**) morphology of the fully deployed occluder device; (**C**) successful puncture of the interatrial septum through the puncture site reserved by the occluder device after 3 months of initial implantation; (**D**) left atrial angiography performed through the sheath inserted via the interatrial septum; (**E,F**) no residual shunting observed immediately after the implantation of the occluder device; (**G,H**) cardiac ultrasound demonstrating the morphology of the occluder device after implantation.

After 3 months of follow-up, the experimental pigs with implanted PFO occluder devices underwent a repeat interatrial septal puncture procedure using a novel occluder device. The puncture needle was aimed at the reserved area on the occluder device. When the sheath was advanced into the reserved puncture hole, the puncture needle was pushed forward, resulting in a breakthrough sensation and pressure monitoring, indicating a successful puncture into the left atrium. A contrast agent was injected to confirm the successful puncture, as observed by the dispersion of the contrast agent within the left atrium (Figure 2).

### The morphology and properties

After three months, the occluder was removed to observe its gross morphology. Tissues surrounding the occluder were collected for pathological examination. Subsequently, the occluder was embedded in methyl methacrylate, sliced using a diamond knife, stained, and observed for gross morphology and histological changes using light microscopy and scanning electron microscopy.

## Results

The PFO model was successfully established in all model pigs with a PFO diameter of (3.56 ± 0.25 mm). The occlusion of PFO using the reserved area of the interatrial septum for occluder placement was successful in all animal models. Confirmation by transesophageal echocardiography showed no residual shunting after PFO closure. During the follow-up period, no deaths or complications such as residual shunting, occluder detachment, or device-related thrombus formation occurred. All implanted occluders remained stable with optimal shape. Left ventricular ejection fraction and cardiac structure were within normal range. Interatrial septal puncture procedures were performed using the occluder in all pigs implanted with the novel occluder. All experimental pigs were euthanized after undergoing the atrial septal puncture experiment using the new occluder.

**Figure 3:**
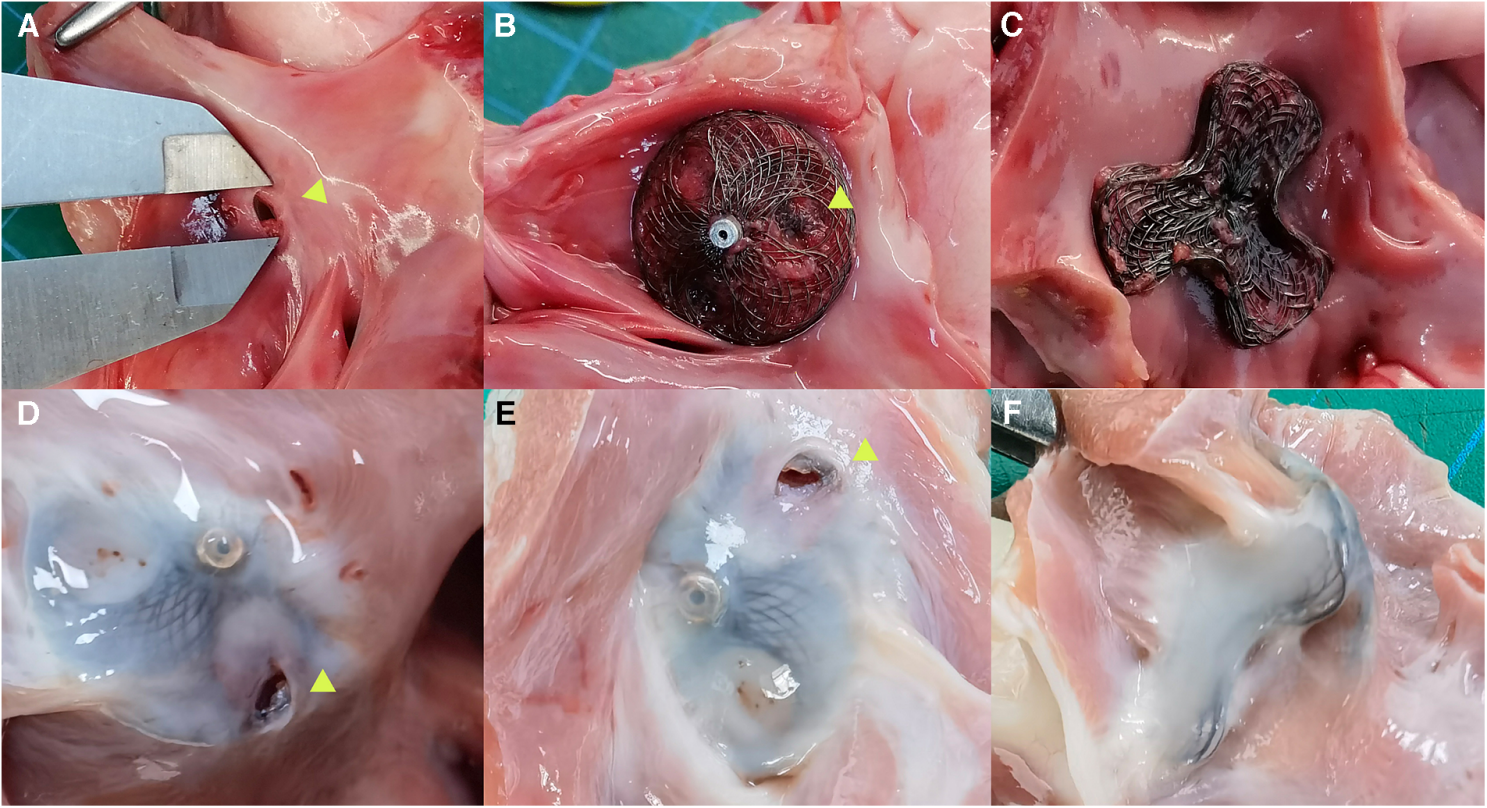
Anatomy specimens of bilateral asymmetrical single-rivet PFO occluder implanted in the reserved area of the interatrial septum. (**A**) Artificial PFO; (**B**) anatomy specimen of the occluder immediately after interatrial septal puncture, shown as a right atrial side view plate with an arrow indicating the interatrial septal puncture hole; (**C**) left atrial side view plate of the occluder immediately after implantation, closely attached to the interatrial septum; (**D,E**) anatomy specimens of the occluder taken 3 months after implantation and subsequent interatrial septal puncture, shown as right atrial side view plates with a gray-white surface covering the internal membrane, with an arrow indicating the interatrial septal puncture hole; (**F**) anatomy specimen of the occluder taken 3 months after implantation, shown as a left atrial side view plate with a completely covered internal membrane surface.

The pathological specimens of the occluder immediately after implantation showed intact occluder structure, without any evidence of wire fracture or overall damage to the occluder. The bilateral disks were closely attached to the interatrial septum, and no thrombus formation was observed on the occluder surface. Pathological specimens of the PFO occluder were obtained after 3 months follow-up. Interatrial septal puncture revealed a layer of gray, smooth, semi-translucent tissue covering both sides of the disks. A puncture hole was visible in the reserved puncture area on the right atrial side, without any other observed damage in the cardiac chambers, including the left atrial cavity or pericardial obstruction (Figure 3).

**Figure 4:**
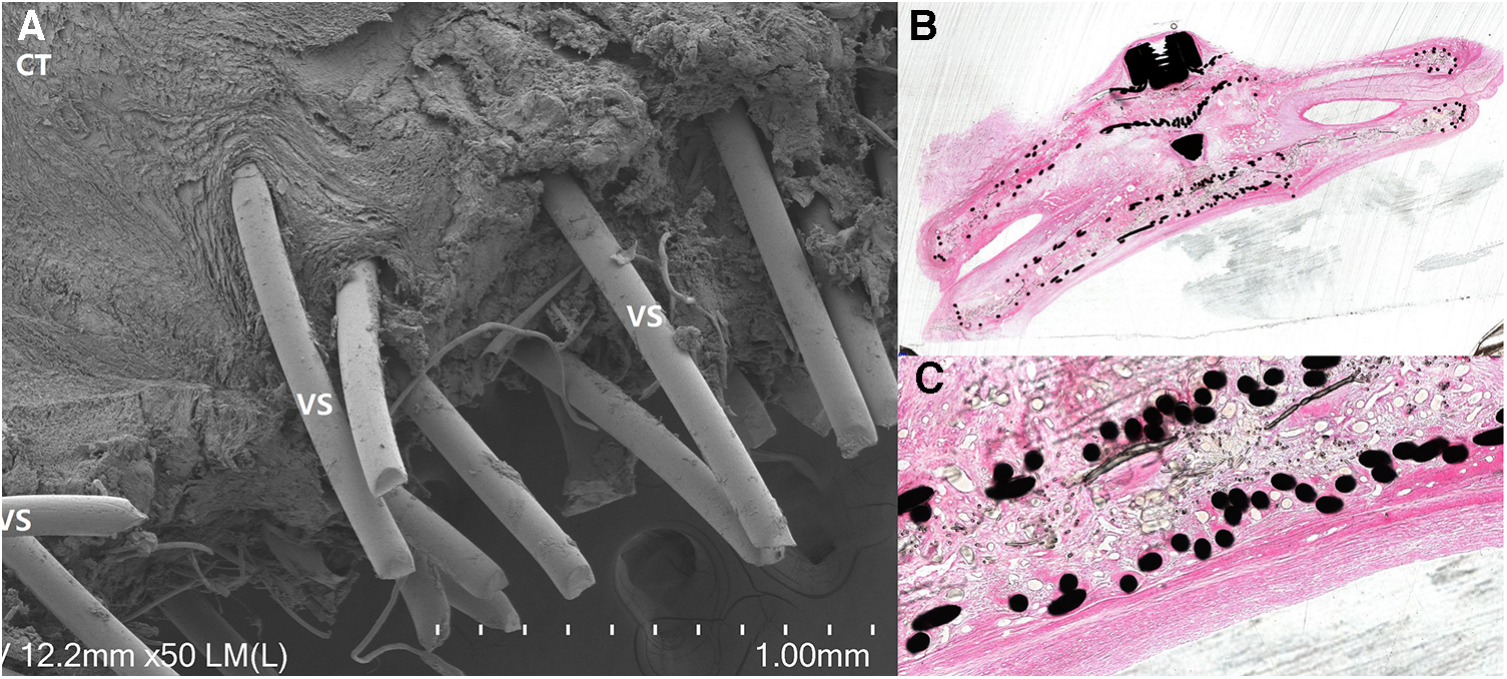
Pathological and electron microscopy specimens: (**A**) electron microscopy specimen revealed the presence of surrounding tissue covering the nitinol wire, with a significant amount of connective tissue (CT) filling the material gaps or surrounding it, demonstrating a tight structure. (**B**) Cross-sectional diamond knife slices demonstrated a well-preserved and structurally intact occluder, with the nitinol wire completely covered by connective tissue and fully enveloped by connective tissue and endothelial layers. (**C**) Neatly arranged nitinol wires, enveloped by connective tissue, with a surface layer consisting of connective tissue and an endothelial layer, appearing smooth and even.

Under optical microscopy, an increase in endothelial cell thickness was observed with prolonged implantation time. After 3 months, a complete layer of endothelial tissue had formed. Scanning electron microscopy revealed minimal flattened cells (C) attachment on the occluder surface, surrounded by connective tissue (CT). Cross-sectional diamond knife slices revealed a well-preserved and structurally intact occluder. The nitinol wire was entirely enveloped by connective tissue and endothelial layers, resulting in a smooth and uniform surface across the entire occluder structure (Figure 4).

## Discussion

PFO is a subtype of interatrial septal defects, where the primary and secondary septa fail to close when an infant begins to breathe (1). The prevalence of PFO in the general population ranges from 20% to 30% (2, 3), with autopsy findings showing a rate of 27% (4). In the largest epidemiological transcranial Doppler (TCD) study conducted on a Greek population, nearly 43% of healthy young individuals had PFO regardless of the shunt size, making it the highest reported prevalence so far. Moreover, there have also been significant variations in the prevalence of PFO observed in transesophageal echocardiography (TEE) studies, ranging from as low as 11% in individuals under 55 years of age to as high as 43% (5, 6).

For patients experiencing cryptogenic ischemic stroke, percutaneous closure of PFO has emerged as a primary treatment for preventing recurrent ischemic stroke (7, 8). Long-term follow-up studies have shown that compared to medical therapy alone, transcatheter closure of PFO is associated with a lower risk of recurrent ischemic stroke in patients with cryptogenic stroke (9). However, subsequent interatrial septal puncture after device implantation in the interatrial septum becomes challenging. With the global aging population and improved survival rates of chronic diseases, the incidence and prevalence of cardiovascular diseases such as atrial fibrillation, valvular disease, and heart failure in older individuals continue to rise (10–12). For instance, atrial fibrillation (AF) is experiencing an increasing incidence and prevalence worldwide. The FHS (Framingham Heart Study) data showed that the prevalence of atrial fibrillation (AF) has tripled in the past 50 years. The Global Burden of Disease project estimated that in 2016, approximately 46.3 million people worldwide had AF (13). In recent years, percutaneous left atrial appendage closure (LAAC) surgery has become an effective procedure for stroke prevention. Pulmonary vein isolation (PVI) performed via interatrial septal puncture is now considered the cornerstone of catheter-based treatment for paroxysmal and early persistent atrial fibrillation (14, 15). Mitral regurgitation (MR) is a common valvular disease (16). The MitraClip device is a form of transcatheter mitral valve repair that can be considered for patients who are not suitable for surgery or have a high surgical risk (17). Interventional treatments such as percutaneous left atrial appendage closure (LAAC) and transcatheter mitral valve repair often require interatrial septal puncture. However, after implantation of a septal occluder, it becomes difficult to successfully perform interatrial septal puncture and proceed with the next surgical steps.

In the past, there have been reports of left atrial side surgery following the implantation of occluders through a punctured atrial septum. Fitzpatrick et al. reported cases of catheter ablation for arrhythmias performed through the punctured atrial septum after occluder implantation in five patients (18). In one case, a 47-year-old male patient attempted to puncture through the center rivet of the occluder using an SJM BRK-1XS Brockenbrough needle (Abbott) through an 8-F SL0 sheath following the conventional approach. Despite continuous balloon dilation, the procedure was unsuccessful as the mesh structure of the occluder would recoil and hinder the passage of the delivery sheath once the balloon was deflated. They then chose to puncture away from the central rivet where the nickel-titanium wires were less dense, achieving success. Two other patients also achieved successful punctures from the edge of the occluder, including one female patient who underwent CT and TEE evaluations and successfully punctured the portion of the atrial septum not covered by the occluder. Worth mentioning is a 59-year-old female patient who had undergone interventional closure of an atrial septal defect 15 years prior. During the initial puncture attempt, the force required for the puncture distorted the atrial septum, potentially leading to posterior perforation or device embolization, resulting in an initial failed puncture.

Fitzpatrick et al. proposed that CT imaging analysis, transesophageal echocardiography (TEE), and intraoperative digital subtraction angiography (DSA) are highly important in these procedures. Performing such complex punctures exposes patients to a higher risk of complications, such as inadvertent puncture into the aorta, penetration into the pericardial space through the posterior wall, and occluder-related risks, such as embolization. This procedure carries a higher risk for the operator as well. Although technically feasible, this complex surgical approach should be avoided whenever possible. Pedersen et al. reported a case of a 37-year-old female who developed atrial arrhythmia after undergoing percutaneous closure of the acquired atrial septal defect using the Amplatzer ventricular septal occluder (19). The arrhythmia was successfully ablated through trans-diaphragmatic puncture guided by esophageal guidance. In the future, there may be an increased demand for left atrial access after device implantation.

To address this issue, we have developed various PFO (patent foramen ovale) occluders allowing interatrial septal puncture after implantation. The first type is a bilaterally symmetric occluder proven effective and safe in previous studies. Building upon this foundation, we have further improved and innovated by developing a bilaterally asymmetric disc occluder that enables interatrial septal puncture after implantation. Our developed asymmetric PFO occluder with preserved puncture area clearly visualizes the tantalum markers at the puncture site and its boundaries under DSA fluoroscopy, effectively guiding the puncture procedure. The puncture area is located on the interatrial septum, eliminating additional CT and TEE examinations to confirm the puncture position. Furthermore, there is no metallic mesh within the puncture area, allowing for easy passage of the puncture needle through the atrial septum without excessive force. This avoids the risks associated with excessive puncture force, such as septal distortion, posterior perforation, or occluder embolization.

Currently, the dimensions of the new occluder are as follows: 18–40 mm. This means that the diameter of the left atrial disk is 18 mm, the diameter of the right atrial disk is 40 mm, the waist diameter is 0.11 mm, and the height is 4 mm. The diameter of the puncture hole is 8 mm, allowing for delivery through a 9Fr sheath. As we are currently in the animal experimentation phase, only one model size of the occluder has been manufactured. In the future, we will produce models ranging from 18 to 30 mm in size.

In three randomized controlled trials, using different double-disc permanent implant devices for percutaneous closure of patent foramen ovale (PFO) has been proven safe and effective (20–22). Double-disc occluders provide reliable closure and have minimal residual shunting through the PFO. Rubine et al. found that in the RESPECT study, with the use of the Amplatzer PFO occluder, the incidence of atrial fibrillation was only 4% during a median follow-up of 5.9 years (23). In a study of PFO closure in 1,000 European patients, there were no thrombotic events with the Amplatzer device, while the incidence of thrombotic events with the CardioSEAL device was 7.1% (24). The Cardioform device has been reported to have some device-related thrombus formation (25, 26). The Figulla Flex II PFO occluder is also a self-expanding double-disc implant made of nitinol wire with superelastic characteristics. Daniela et al. treated 442 patients with the Figulla Flex II device (Occlutech, Germany) for PFO closure and followed them for 10 years. One case of device embolization resulted in treatment failure, and there were 15 in-hospital complications (3.4%; 4 cases of minor access site complications, 11 cases of transient supraventricular tachycardia/atrial fibrillation). At a follow-up of 9.2 years, two patients had recurrent transient ischemic attacks (without residual right-to-left shunt), and three patients showed moderate or more significant residual shunting upon discharge (27).

Compared to traditional PFO occluders, the novel PFO occluder has unique morphology and weaving methods. The right disc of the occluder is a conventional circular disc shape, with a degradable membrane that blocks the blood flow. It includes a pre-reserved puncture hole on the right atrial side. In contrast, the left disc is in a “cloverleaf” shape with an extremely dense weaving structure. The PFO occluder is primarily designed to prevent right-to-left shunting. This design has been adapted accordingly. The right disc is mainly utilized for blocking the shunt, while the left disc is primarily used for fixation. In our experiments, all occluders completely sealed the PFO model, with no residual shunting observed. Furthermore, the novel occluder has a visible marker in the puncture area for future puncture procedures under DSA (digital subtraction angiography) guidance.

Previous studies have found that after implantation of a PFO occluder, there is an increased incidence of new-onset atrial fibrillation (AF) and other arrhythmias in patients. Compared to the medication group, patients who underwent transcatheter PFO closure surgery have a 5.3-fold higher risk of developing atrial fibrillation. This increased risk of AF is concentrated in the first 45 days after the procedure and may be persistent or intermittent (28). This phenomenon may be related to the pressure exerted on the atrial septum by the occluder. The surgical process can cause atrial stimulation, and the occluder may trigger an inflammatory response or act as a mechanical barrier, creating a large loopback pathway (29, 30). Our novel occluder significantly reduces the metallic portion of the double discs, resulting in a reduced coverage area of nitinol wire on the atrial septum. The right disc of the occluder has a degradable membrane covering the reserved puncture area, while the left disc takes on a unique “cloverleaf” shape with a tighter structure and less contact area with the atrial septum tissue, thus reducing tissue compression. Consequently, this reduces tissue edema, inflammation, and fibrosis, leading to a decrease in the occurrence of arrhythmias.

Artificial PFOs are created by puncturing the atrial septum and establishing a wire-guided pathway, through which a high-pressure balloon with a diameter of 5 mm is inserted and inflated to a pressure of 24 atmospheres. This type of PFO has a straight channel with irregular edges. On the other hand, natural PFOs occur due to improper adhesion between the primary and secondary atrial septa during development. The diameter of the natural PFO is smaller, with a curved and oblique or horseshoe-shaped pathway, typically measuring less than 10 mm in length. The length of a long-tunnel PFO can reach up to 20 mm. In theory, the stability of occluding artificial PFOs using the new occluder may be relatively poorer due to their smaller diameter (around 3 mm) and shorter length. However, in our study, we did not observe any instability or residual shunting after occluding artificial PFOs with the new occluder. Based on this, we speculate that the new occluder can effectively be used for PFO closure.

The research findings indicate that the new occluder can effectively close the PFO model without a residual shunt. Postoperatively, no movement of the occluder position was observed. Specimens from the animals sacrificed three months later demonstrated complete endothelial coverage over the occluder, intact occluder structure, absence of thrombus formation, and no apparent nickel-titanium wire fracture. Three months after occluder implantation, interatrial septal puncture within the preserved area of the interatrial septum was performed. It is worth mentioning that all surgical operations were executed efficiently and demonstrated considerable enhancement in performance when compared to the first-generation occluder.

## Conclusions

Utilizing a bilateral asymmetric single-rivet occluder device implanted through the reserved interatrial septal puncture area has proven effective in closing PFO. After implantation, the occluder device allows subsequent interatrial septal puncture procedures through the reserved area. The novel occluder device demonstrated excellent closure performance, biocompatibility, and puncturability in the experiment. This indicates the feasibility of conducting further catheter-based interventions on the interatrial septum.

### Limitations of the study

Firstly, our study had a small sample size and a relatively short follow-up period. Further research with larger sample sizes and longer follow-up times is needed to obtain sufficient data for potential clinical applications. Secondly, we did not establish a control group with conventional patent foramen ovale (PFO) closure. Thirdly, the tricuspid leaflet-like disc on the left atrial surface has a different stiffness compared to conventional occluders. It is important to investigate whether there are any gaps when it adheres to the atrial septum and to study further thrombus-related issues associated with the device.

The PFO model we used was also created through balloon dilation, resulting in irregular edges and shallow depths. In contrast, a natural PFO exists due to incomplete fusion between the primary and secondary atrial septa, often presenting as an oblique or even horseshoe-shaped pathway. There are morphological differences between our model and a natural PFO that should be considered.

In future studies, we will compare the different performances of occluders of various sizes.

## Data Availability

The datasets presented in this study can be found in online repositories. The names of the repository/repositories and accession number(s) can be found in the article/Supplementary Material.
